# Sero‐detection of *Coxiella burnetii* infection in cattle, sheep and goats in selected regions of Nepal

**DOI:** 10.1002/vms3.458

**Published:** 2021-03-01

**Authors:** Narayan Paudyal, Subash Poudel, Durga Pandey, Doj R. Khanal

**Affiliations:** ^1^ National Animal Health Research Centre NARC Lalitpur Nepal; ^2^ Muna Veterinary Hospital Lalitpur Nepal; ^3^ Himalayan College of Agricultural Sciences & Technology Kirtipur Kathmandu Nepal

**Keywords:** coxiellosis, goat, livestock, nepal, prevalence

## Abstract

*Coxiella burnetii*, a Gram‐negative bacterium is a zoonotic agent causing coxiellosis in animals. Small ruminants and cattle are the primary reservoirs for human infection. This study was aimed to estimate the sero‐prevalence of *C. burnetii* in the ruminants of the selected region in Nepal. Field visits were carried out at four sites in different geographical regions of Nepal. A total of 522 sera samples were collected from 118 sheep, 242 goats and 162 cattle with the history of abortion, anoestrus and infertility. Sera were tested for the presence of antibodies against *C. burnetii* using a commercially available ready‐to‐use ELISA test kit. The overall true sero‐prevalence was 1.89% (95% CI: 0.33–3.45), the prevalence ranged between 4.35% and 23.21% in goats. Sero‐prevalence in goat was higher than that of cattle and sheep which ascertained that total freedom from coxiellosis cannot be confirmed in Nepal. This could complement the impacts of other infectious causes of the infertility in the farm animals as well as the public health of the farming households.

## Graphical abstract

The figure shows the geographic distribution of the sampled sites in a map of Nepal.

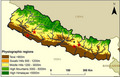

**Impacts**

- Coxiellosis is not officially reported in Nepalese livestock yet.
- We report the presence of antibodies against the pathogens in farm livestock (in absence of vaccination).
- This signifies that the disease is circulating in livestock in Nepal where presence in humans has already been reported.

## 1 INTRODUCTION

Coxiellosis (referred to as Q‐fever in humans) is a zoonotic disease caused by *Coxiella burnetii*, a Gram‐negative and obligate intracellular bacterium (van der Hoek et al., 2012). The febrile illness “Query fever” (Q‐fever) was first reported in 1935, among workers of a slaughterhouse in Australia (Gwida et al., 2012). Several domestic and wild animals, as well as birds, reptiles and arthropods (ticks), can harbour the pathogen (Ullah et al., 2019). Cattle, goats and sheep are the main reservoirs where most of the infection is asymptomatic, but abortions or stillbirths may occur (Pexara et al., 2018). Being viewed as an occupational hazard of humans working in proximity to animals (Psaroulaki et al., 2006), the zoonotic infection has aroused interest among the researchers after the surge in human infections in the Netherlands during the years 2007–2009 (Vanderburg et al., 2014). In Nepal, multiple cases of undifferentiated febrile illness in humans were later on found to be positive for Q‐fever in 2015 (Thompson et al., 2015). Similarly, a recent study conducted on 104 cattle sera samples collected from Chitwan, Nawalpur and Rupandehi districts of Nepal had shown the absence of the antibodies against *C. burnetii* (Shrestha & Singh, 2020). But another study, also conducted in the Rupandehi district on 184 cattle sera samples showed an apparent sero‐prevalence of 1.63% (Panth et al., 2017).

The infected animal hosts shed the pathogens in faeces, urine, milk, placental membranes as well as the amniotic fluid (Park et al., 2018). This pathogen has been reported to be able to survive outside of the host for a protracted period and human outbreaks can also result from the inhalation of aerosolized organisms (Arricau‐Bouvery & Rodolakis, 2005).

Serological surveys have been performed in many countries to evaluate the distribution of *C. burnetii* in domestic ruminants. Routine diagnosis of Q‐fever is established by serological tests that include immunofluorescence assays (IFAs), complement fixation test CFT) and enzyme‐linked immunosorbent assays (ELISA). Among these, ELISA has slightly higher sensitivity as compared with IFA for use in small ruminants but similar data on large ruminants are unavailable (Rousset et al., 2007). A study conducted in 2016 has concluded that IFA is better suited as compared with the CFT or ELISA for detecting the IgG and IgM in the serum of goats (Muleme et al., 2016).

A study on bovine infertility that has been undergoing for the last 4 years in Nepal has shown that there is the co‐occurrence of sub‐clinical mastitis, brucellosis and some mineral deficiency in the majority (approximately > 85%) of the cases of decreased fertility (unpublished data of the corresponding author). However, the impact of *C. burnetii* in bovine infertility in Nepalese context has not been evaluated, despite its well‐recognized role in decreased fertility or infertility in farm animals globally (de Oliveira et al., 2018; Pexara et al., 2018). Therefore, this study was set up to evaluate the sero‐prevalence of *C. burnetii* in infertile bovines and small ruminants and establish a baseline data on the possible presence of this pathogen, which has not yet been officially reported in Nepal.

## 2 MATERIALS AND METHODS

Four districts (two from western Nepal and two from central Nepal) were selected for sampling. These sites were the same sites where previous work on the nationally coordinated project on bovine infertility had been implemented. The farms and animals which had reported some incidence of issues related to infertility in the last year were purposively selected. The farms were located with the data provided by the respective district livestock service offices. A structured questionnaire was used to record allied meta‐data including multiple identifiers such as the name of the owner (villages), species, breed, sex, estimated age, stage or lactating and apparent health condition. A total of 522 blood samples (118 sheep samples, 242 goat samples and 162 cattle samples) were collected. The individual household was considered as a sampling unit for the sampling purpose. Due to the integrated livestock husbandry system of rural Nepalese farming household, we could collect samples of goat, cattle and sheep from the same household but in varying numbers. While there were 168 households included in our sampling, it included only 18 organized dairy farm (cattle) and 11 goat farms and 3 sheep farms, only. In all dairy cattle farms (organized or household), the primary method of breeding was the artificial insemination (AI), however, on repeated failure of AI (typically more than four times), the farmers opted for natural mating. In the case of sheep and goats, there was no practice of AI; all the farms and households practised natural service with breeding bucks. The primary forms of infertility as mentioned by the farmers were repeat breeding and abortion.

The sample size was calculated in EpiTools (Sergeant, 2018). We used the tool to estimate “sample size for apparent or sero‐prevalence” with an estimated proportion of 0.5, desired precision of the estimate as 0.05 and a confidence level of 0.95 in population size of 300,000. The suggested minimum sample size was 385. During the sample collection, the farmers were informed that their animals were undergoing evaluation for some diseases of zoonotic importance. During the evaluation process, farmers insisted that we collect samples from their animals which we could not deny due to practical reason thus the sample size increased to 522.

The blood sample was collected by jugular vein puncture, and serum was separated using a portable centrifuge. All sera samples were transported on ice to the immunology and serology laboratory of the National Animal Health Research Centre and stored at − 20^0^C until tested for antibodies at a later time. None of the animals used for sample collection had a history of vaccination against coxiellosis.

### 2.1 Serology

A commercial ELISA Kit (IDscreen^®^Q fever indirect multi‐species, IDvet France) was used. As provided by the manufacturer, the sensitivity of the ELISA was 100% (95% CI: 89.28%–100%) and the specificity was 100% (95% CI: 97.75%–100%) (Changoluisa et al., 2019). The calculation of 95% CI for the apparent as well as true prevalence was based on the normal approximation method. The manufacturer's protocol was followed without any manipulations. Each sample was tested in duplicate. As suggested, the plates were read at 450 nm in 96 well‐plate capacity laboratory spectrophotometer (Ledetect 96). Only the tests that met the validity criteria as suggested in the kit was considered valid. Plans were put in place to re‐analyse any doubtful samples for once and excluded if remained doubtful. The optical densities (ODs) of samples were analysed using the negative and the positive controls using the recommended formula.

$$S/P\left(\%\right)=\frac{\text{ODsample}‐\text{ODneg}}{\text{ODpos}‐\text{ODneg}}\times 100.$$

S/P (%) value of more than 80% was considered as strong positive; a value between 50% and 80% was considered positive; a value between 40% and 50% was considered doubtful and less than 40% was regarded and analysed as negative. Other statistical analyses were done on GraphPad Prism versus 8 on a Windows platform. We used two‐way ANOVA to compare the prevalence of diseases among the districts and animals (called as row factor) and the animal species (called as column factor) in GraphPad Prism versus 8.

## 3 RESULTS

A total of 522 serum were obtained from sheep, goats and cattle from four districts of Banke (*n* = 238) and Surkhet (*n* = 185), Kavre (*n* = 92) and Makawanpur (*n* = 7), the details of which are given in Table 1. Among these 522 total sample, only 14 samples were found to be seropositive to the antibodies against *C. burnetii*. None of the samples yielded doubtful results. Based on this outcome, the apparent and true prevalence was calculated (Table 2).

**TABLE 1:** Sample distribution according to the sites

SN	District	Animal	Total	Positives
1	Surkhet	Sheep	64	4
		Goat	51	0
		Cattle	70	2
2	Banke	Sheep	54	0
		Goat	92	0
		Cattle	92	0
3	Kavre	Goat	92	6
4	Makwanpur	Goat	7	2
Total			522	14

**TABLE 2:** Details of the prevalence (95% CI) in animals at different sites

SN	Site	Animal	Apparent prevalence (95% CI)	True prevalence (95% CI)
1	Surkhet	Sheep	6.25 (0.32, 12.18)	−4.69 (−12.1, 2.73)
2	Surkhet	Cattle	2.86 (−1.05, 2.73)	−8.93 (−13.81, −4.05)
3	Kavre	Goat	6.52 (1.48, 11.57)	4.35 (−10.65, −1.96)
4	Makawanpur	Goat	28.57 (4.89, 62.04)	23.21 (−18.62, 65.1)
Total			2.68 (1.3, 4.07)	1.89 (0.33, 3.45)

**FIGURE 1:**
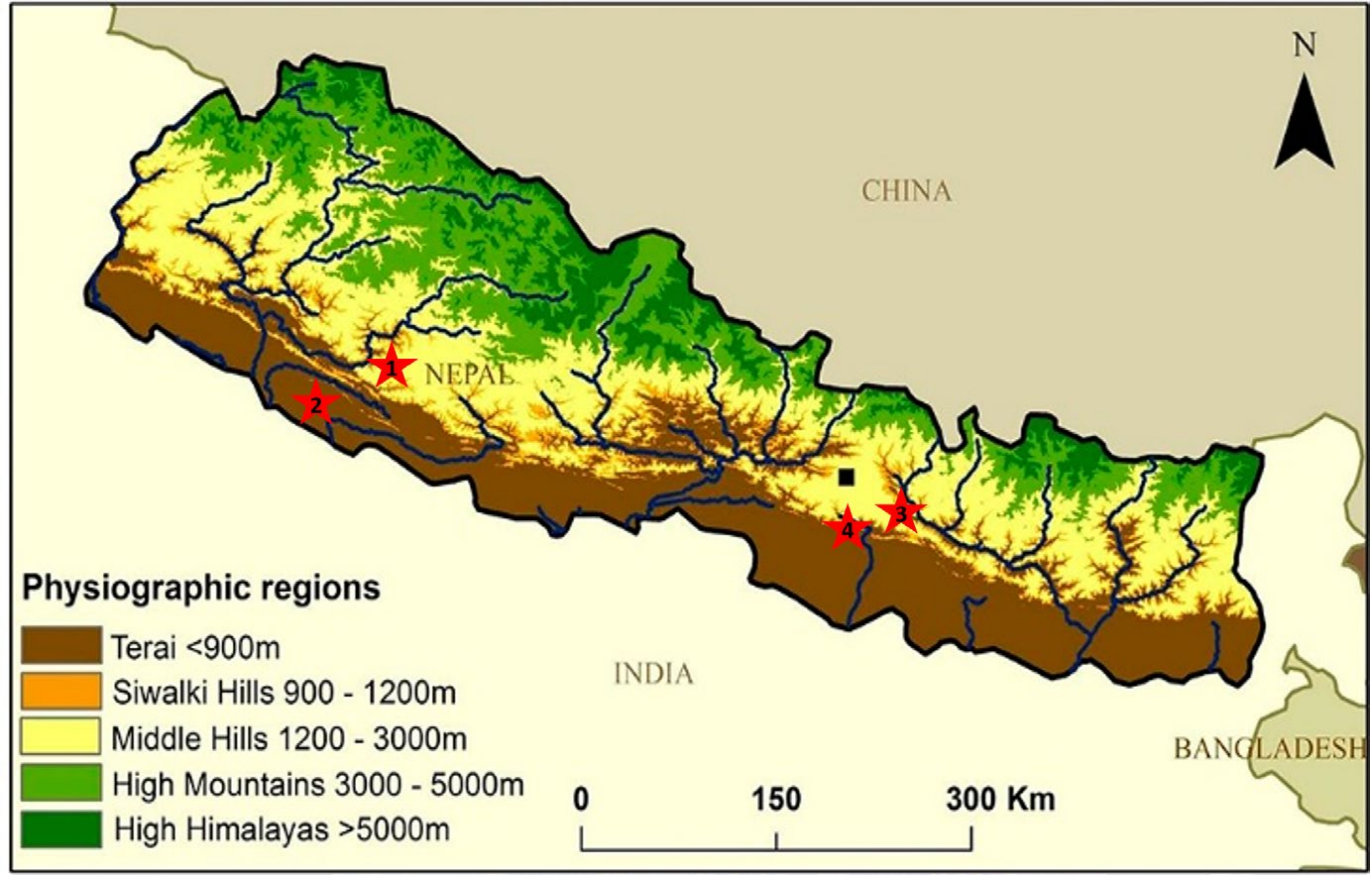
Schematic representation of the four different sites of sampling. The sites are numbered in line with that of Table 1. The basic figure has been sourced from earthwise.bgs.ac.uk/images/1/15/OR14069fig1.jpg

Apparent prevalence in sheep was higher than in cattle but it was the highest in goat, so was the true prevalence. The true prevalence was negative for both animal types (sheep and cattle) at Surkhet but positive at Kavre and Makawanpur. It is possible that the true prevalence of coxiellosis at Surkhet was beyond the minimum detection level of the ELISA used. No antibodies were detected in any sera from Banke district (Figure 1).

The apparent prevalence of Q‐fever in sheep at Surkhet was 6.25% (95% CI: 0.32 – 12.18) [true prevalence of −4.69%, 95% CI: −12.1 – 2.73] and in cattle was 2.86% (95% CI: −1.05–2.73) [true prevalence of −8.93%, 95% CI: −13.81‐ −4.05]. Similarly, in the goats of Kavre, the apparent prevalence was 6.52% (95% CI: 1.48–11.57) [true prevalence −4.35%, 95% CI: −10.65‐ −1.96] and that in goats of Makawanpur was 28.57% (95% CI: −4.89–62.04) [true prevalence of 23.21, 95% CI: −18.62–65.05]. The two‐way ANOVA analysis showed that in the positive results, the variation contributed by geographic region was 20.33% and that contributed by the animal species was 21.10%, both of which were statistically non‐significant.

Most of the cattle populations sampled were of the mixed breed type so the association of breed to the sero‐positivity could not be ascertained, similar was the case with the sheep where most of the population was local sheep. But in the case of goats, the sero‐positivity of the indigenous breed was approximately 5% while that of the exotic cross breeds was 7.6%. There was no statistical significance (*p* = 0.604) in the difference between the types of breed for sero‐positivity to coxiellosis. Similarly, sero‐positivity was more in older animals (>3 years) but it also was statistically non‐significant.

## 4 DISCUSSION

To the authors’ knowledge, this is the first sero‐prevalence study of *C. burnetii* in sheep and goats of Nepal, although one earlier study has reported the sero‐positivity in cattle (Panth et al., 2017). This disease is officially not reported in Nepal. A recent literature review on the prevalence of *C. burnetii* infection in domestic ruminants in different countries worldwide revealed a wide variation in reported prevalence and study quality. Of many publications reviewed therein, a research performed in the neighbouring country of India reported 5.4% and 1.85% sero‐prevalence of *C. burnetii* in goats and sheep, respectively, at Pondicherry and Tamil Nadu. Results of our study showed a similar true sero‐prevalence in sheep which suggests that Q‐fever is an emerging infectious disease of the sheep and even for the goats in Nepal. A recent study conducted at Chitwan, Nawalpur and Rupandehi districts of Nepal, on 104 cattle samples had shown the absence of the antibodies to *C. burnetii* (Shrestha & Singh, 2020). But another study also conducted in the Rupandehi district on 184 cattle sera samples showed an apparent sero‐prevalence of 1.63% (Panth et al., 2017).

The present investigation revealed that the true prevalence of coxiellosis in sheep of Surkhet was negative but positive at a 95% confidence interval was positive for only the prevalence of sheep (2.73%). If a test with less than 100% sensitivity and specificity is used to estimate the prevalence of some characteristic, that estimate will invariably be biased. It is also accepted that no test is perfect (as determined by the sensitivity and specificity), and given the test parameters for this particular commercial ELISA vary in a wide range (sensitivity of 89.28%–100% and specificity of 97.75%–100%) (Changoluisa et al., 2019), it is plausible that the very low prevalence could not be correctly detected by this test but the negative ones could have been estimated correctly. This shows that we cannot rule out the absence of coxiellosis in farm ruminants of Nepal but the prevalence could be too low to be accurately detected by commercially available ELISA tests. Additionally, the absence of antibodies in the serum of goats in some region could be possibly due to the fragmented and compartmentalized distribution of this pathogen.

As seen in our study, sero‐prevalence in goats and sheep is generally regarded to be higher than in cattle (Pexara et al., 2018; Van den Brom et al., 2015) but these vary. Globally, different rates of prevalence have been reported in livestock populations. In a study from Switzerland, a low level of sero‐positivity at 3.4% for goats and 1.8% for sheep was reported (Magouras et al., 2017). A Greek study showed the sero‐prevalence to be 14.4% for goats and 8% for sheep (Filioussis et al., 2017). In Iran, positivity rates were 27.2% for goats and 19.5% for sheep (Asadi et al., 2013). In the Punjab region of India, the overall individual animal prevalence of coxiellosis was estimated at 7.0% (95% CI: 4.7–9.4) (Keshavamurthy et al., 2019).

Regarding its role in infertility, coxiellosis was not an important cause for abortion in cattle in Ecuador (Changoluisa et al., 2019). In most animals, the infection is asymptomatic, but abortions or stillbirths can occur (de Oliveira et al., 2018). In our analysis, we did not find any statistically significant correlation. However, this does not imply that there may not be any biological association, the evaluation of which was beyond the scope of this study. It was also interesting to note than in the goat samples of Makawanpur, all the positive samples were obtained from goats crossbred with Boer or Saanen while the indigenous goats were entirely negative. So in future, it would be more inclusive to enlist coxiellosis in the differential diagnosis of the investigations on the infective causes of abortion in animals of Nepal. There is a study which showed that brucellosis and coxiellosis co‐exist with high prevalence in female cattle in Nigeria (Adamu et al., 2018).

Although the results of other descriptive analysis, such as age and the breed, were statistically non‐significant, this could have biological significance. For example, older animals can have developed a carrier state without any symptoms (Pexara et al., 2018). Similarly, the results of association of breed and sero‐positivity were statistically non‐significant. An old study carried out in Canada (Hatchette et al., 2001) reports that in humans 40.6% (13/32) were infected with *C. burnetii* showing symptoms of loss of libido. Some field veterinarians in Nepal also reported the absence of libido in bucks of imported exotic goat breeds such as Saanen and Boer, starting 3 years ago. Considering this and the likely endemicity of *C. burnetii*, more detailed surveillance activities to address the problems, such as lack of libido in breeding bucks of imported Boer and Saanen breeds that may not have had earlier exposures to coxiellosis, are warranted. It is well recognized that vaccination is a primary cause of sero‐positivity towards any ELISA test done for disease diagnosis. However, in Nepal, there is no practice of vaccination against this. A 2020 study from India also reported the sero‐positivity towards coxiellosis in dairy herds despite the absence of vaccination against coxiellosis in cattle (Dhaka et al., 2020). Therefore, we infer that our farm animals have had earlier exposures to the pathogen.

Despite this study being the first of its kind for Nepal, there are some limitations. For example, as sampling was not uniform in these selected areas, statistical analyses have been hampered. Furthermore, the sampling was purposive and we could not provide results on the direct testing on the pathogen. Lastly, the farm metadata were not taken for the purpose so the analysis could not be made on those variables such as farm size, farm clustering, farming type and size of the herds investigated.

In conclusion, confirming a very low prevalence, we cannot ascertain the freedom of *C*. *burnetii* in goats, sheep and cattle in Nepal. This might have been contributing to the problems of infertility in such livestock populations. Detailed studies in future on the possible risk factors and their roles in such re‐emerging zoonotic diseases could be beneficial. This eventually sheds light on the importance of “One‐Health” concept to mitigating these diseases at the animal–human interface.

## AUTHOR CONTRIBUTION

**Narayan Paudyal:** Investigation; Methodology; Writing‐original draft; Writing‐review & editing. **Subash Poudel:** Investigation. **Durga Pandey:** Investigation. **Doj Raj Khanal:** Conceptualization; Funding acquisition; Project administration; Resources; Supervision; Writing‐review & editing.

### PEER REVIEW

The peer review history for this article is available at https://publons.com/publon/10.1002/vms3.458.
